# Sleep and circadian disruption reshape immune homeostasis in oral tissues: mechanistic roles of melatonin and cortisol in periodontal disease and xerostomia

**DOI:** 10.3389/fimmu.2026.1781288

**Published:** 2026-04-29

**Authors:** Mu-Hsin Chen, Yu-Ching Hung, Wei-Ni Kuo, Hsuan-Yu Hsu, I-Ta Lee, Cheng-Yu Tsai, Arnab Majumdar, Tien-Li Ma

**Affiliations:** 1 School of Dentistry, College of Oral Medicine, Taipei Medical University, Taipei, Taiwan; 2 School of Respiratory Therapy, College of Medicine, Taipei Medical University, Taipei, Taiwan; 3 Division of Pulmonary Medicine, Department of Internal Medicine, Taipei Medical University-Shuang Ho Hospital, New Taipei City, Taiwan; 4 Sleep Center, Taipei Medical University-Shuang Ho Hospital, New Taipei City, Taiwan; 5 Taipei Medical University (TMU) Research Center of Artificial Intelligence in Medicine and Health, Taipei Medical University, Taipei, Taiwan; 6 Research Center of Sleep Medicine, College of Medicine, Taipei Medical University, Taipei, Taiwan; 7 Taipei Medical University (TMU) Research Center of Thoracic Medicine, Taipei Medical University, Taipei, Taiwan; 8 School of Biomedical Engineering, College of Biomedical Engineering, Taipei Medical University, Taipei, Taiwan; 9 Centre for Transport Studies, Department of Civil and Environmental Engineering, Imperial College London, London, United Kingdom; 10 School of Dental Technology, College of Oral Medicine, Taipei Medical University, Taipei, Taiwan

**Keywords:** circadian disruption, cortisol, immune homeostasis, melatonin, oral inflammatory diseases

## Abstract

Sleep disturbance has emerged as a major modifier of immune homeostasis in chronic inflammatory diseases, yet its immunological relevance to oral tissues remains incompletely integrated. Accumulating evidence indicates that sleep and oral inflammatory diseases are linked through coordinated neuroendocrine–immune pathways, with melatonin and cortisol constituting a central circadian hormonal axis that shapes immune balance. Melatonin, predominantly secreted during the nocturnal phase, exerts antioxidant, anti-inflammatory, and immunomodulatory effects that support periodontal immune homeostasis and salivary gland function. In contrast, sleep disruption and chronic activation of the hypothalamic-pituitary-adrenal axis led to dysregulated cortisol secretion, promoting immune suppression, microbial dysbiosis, and progressive periodontal tissue breakdown. Experimental, observational, and clinical studies consistently associate poor sleep quality with suppressed melatonin rhythms, elevated cortisol levels, and increased prevalence of periodontitis and xerostomia, while sleep disorders such as obstructive sleep apnea further exacerbate oral inflammatory vulnerability through both circadian and mechanical mechanisms. Despite these associations, the causal immune pathways linking circadian hormonal dysregulation to oral disease progression remain insufficiently defined. This review synthesizes current mechanistic and clinical evidence to conceptualize the melatonin–cortisol axis as an immunoregulatory bridge between disturbed sleep and oral inflammatory disease, and discusses its implications for biomarker development and immune-informed therapeutic strategies.

## 1 Introduction

Sleep is a fundamental biological process that regulates systemic immune homeostasis through circadian rhythms, endocrine signaling, and coordinated inflammatory control (1). Modern lifestyles characterized by psychosocial stress, shift work, and widespread use of electronic devices have contributed to an alarming rise in sleep disturbances, including insomnia and obstructive sleep apnea (OSA) (2). The consequences of inadequate or fragmented sleep extend beyond neurocognitive impairment and cardiovascular morbidity to encompass immunological dysfunction and endocrine dysregulation (3). Despite these broad systemic effects, the implications of disturbed sleep for oral health have only recently begun to receive sustained attention (4).

Oral diseases such as periodontitis, a chronic inflammatory condition affecting the supporting structures of the teeth, and xerostomia, defined as the subjective sensation of dry mouth often accompanied by hyposalivation and compositional salivary changes, provide unique windows through which the interplay between circadian biology and local tissue homeostasis can be studied (5, 6). From an immunological perspective, oral tissues represent a unique mucosal interface where circadian-regulated endocrine signals intersect with microbial exposure and host immune surveillance. Accumulating evidence indicates that these conditions are not isolated oral problems but rather integral components of systemic inflammatory networks that are strongly influenced by sleep quality (7).

**Figure 1:**
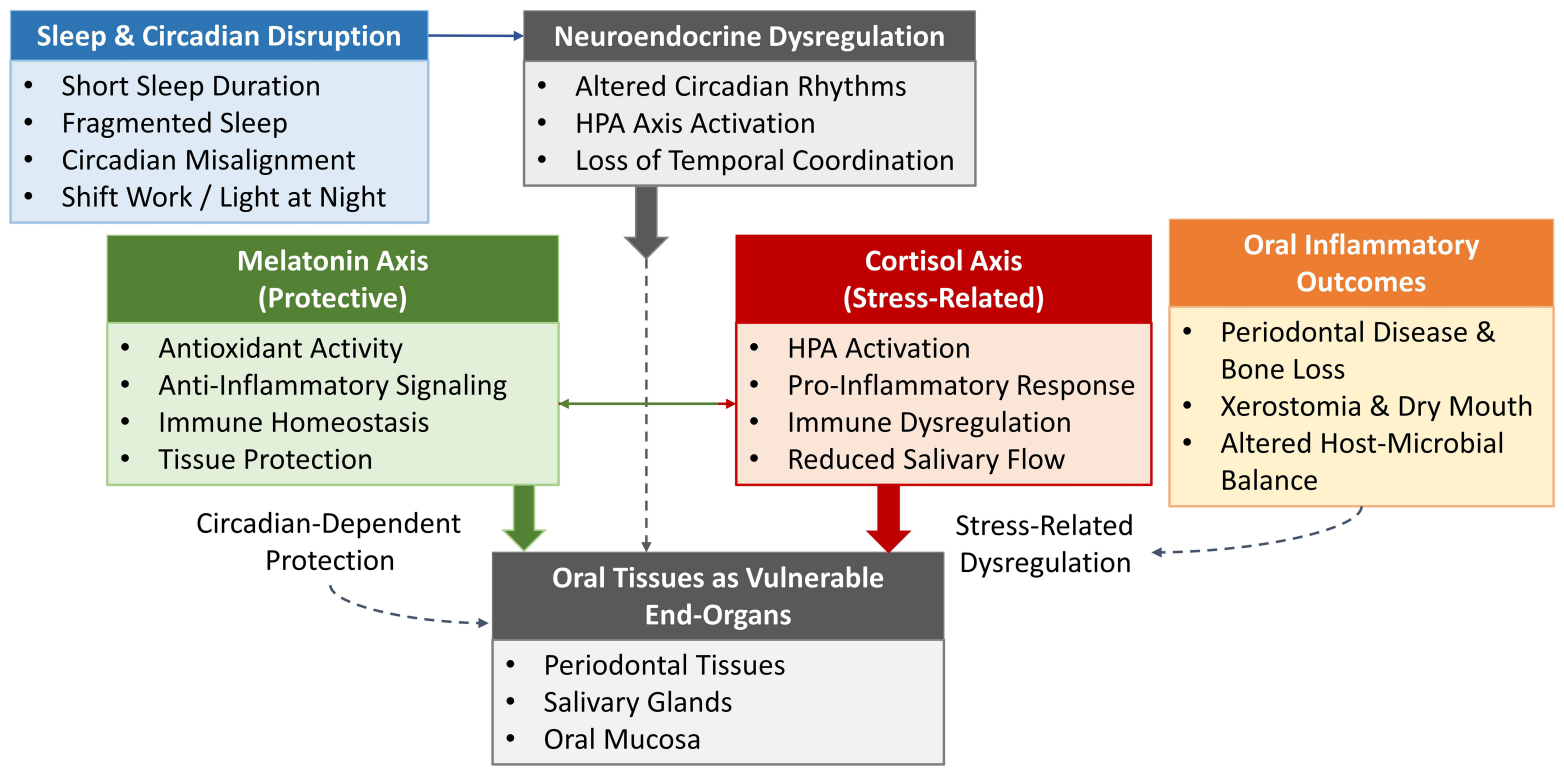
Sleep and circadian disruption induce neuroendocrine imbalance characterized by reduced melatonin signaling and dysregulated cortisol rhythms, rendering oral tissues vulnerable to inflammatory and functional impairment.

Two hormones, melatonin and cortisol, are central mediators in this context. Melatonin, secreted nocturnally by the pineal gland, functions not only as a regulator of circadian rhythms but also as a potent antioxidant and immunomodulator with established protective roles in periodontal and salivary gland tissues (8). In contrast, cortisol, the major glucocorticoid released via the hypothalamic-pituitary-adrenal (HPA) axis, exhibits a diurnal rhythm that can become flattened or phase-shifted in sleep disorders, leading to heightened inflammatory tone, impaired immune surveillance, and microbial dysbiosis (9). Together, these hormonal perturbations provide a plausible mechanistic link between disturbed sleep and oral disease progression. This review synthesizes current experimental, clinical, and epidemiological findings on melatonin and cortisol in sleep-related oral health, highlights emerging biomarker and therapeutic opportunities, and identifies critical gaps that must be addressed to integrate sleep biology into precision oral medicine. In this review, we propose a conceptual framework linking sleep and circadian disruption to oral inflammatory vulnerability through dual neuroendocrine pathways involving melatonin and cortisol (**Figure 1**).

## 2 Sleep and hormonal regulation

### 2.1 Melatonin secretion and circadian control

Melatonin, synthesized primarily by the pineal gland during the dark phase, is a critical regulator of circadian rhythms and sleep, wake cycles (10). Its secretion is tightly controlled by the suprachiasmatic nucleus and suppressed by environmental light exposure, allowing synchronization of internal biological clocks with external day-night cycles (11).

Beyond its chronobiological role, melatonin exhibits diverse physiological functions, including potent antioxidant and immunomodulatory actions (12, 13). Melatonin receptors (MT1 and MT2) are expressed in a wide range of tissues, including the brain, retina, blood vessels, gastrointestinal tract, reproductive organs, and notably, periodontal and salivary tissues (14). MT1 receptors are primarily involved in circadian signaling and cellular homeostasis, whereas MT2 receptors contribute to phase shifting and are increasingly recognized for their role in modulating inflammatory responses in peripheral tissues, including periodontal cells. This distribution provides biological plausibility for melatonin’s involvement in oral homeostasis (15).

Sleep disruption, whether through insomnia, shift work, or circadian misalignment, suppresses nocturnal melatonin secretion and blunts its rhythmicity, potentially impairing host defense against oxidative stress and chronic inflammation (16, 17). Clinical studies further suggest that patients with insomnia or irregular sleep schedules demonstrate markedly reduced nocturnal melatonin, particularly during the midnight to early morning hours (18). While melatonin-based interventions have been explored in experimental and clinical contexts in systemic disorders such as metabolic syndrome and cardiovascular disease, its translational application to oral health remains underexplored (19). A key knowledge gap concerns whether exogenous melatonin can effectively mimic the temporal profile of endogenous secretion in oral tissues and whether targeted delivery to gingival or salivary sites confers therapeutic benefit (20). These considerations highlight melatonin as not only a circadian regulator but also a potential mediator linking disturbed sleep to oral disease pathogenesis (21).

### 2.2 Cortisol circadian profile and HPA axis activation

Cortisol, the primary glucocorticoid produced by the adrenal cortex, is secreted under the regulation of the HPA axis and follows a distinct circadian rhythm. Levels peak within 30–45 minutes after awakening, gradually decline throughout the day, and reach their nadir around midnight. This pattern ensures metabolic adaptation, immune vigilance, and energy mobilization in synchrony with daily activity cycles (22).

Sleep deprivation, shift work, and psychosocial stress can disrupt this rhythm (23), leading to elevated evening cortisol levels, a flattened diurnal profile, or overall hypercortisolism. Such dysregulation reflects chronic HPA axis overactivation and is associated with impaired glucose metabolism, heightened systemic inflammation, and reduced resilience to stressors (24). Importantly, evidence indicates that even in the absence of psychological stress, sleep restriction alone can elevate cortisol secretion, underscoring the independent role of sleep in HPA regulation (25).

In the context of oral health, excess cortisol suppresses protective host immune responses, reduces neutrophil chemotaxis, and promotes microbial imbalance (26). These mechanisms together exacerbate periodontal tissue destruction (27). Salivary cortisol, which can be measured noninvasively, has been consistently correlated with the severity of periodontal disease (28). Despite its promise as a biomarker, comparability across studies is limited by methodological variability (29). Differences in sampling time, assay sensitivity, and stress-related confounders contribute to inconsistent results (30). Standardized protocols and longitudinal research are needed to clarify whether restoring normal circadian rhythmicity, through sleep-based interventions or chronotherapy, can mitigate cortisol-driven oral pathology (31).

### 2.3 Hormonal interaction with immunity and inflammation

The interplay between melatonin and cortisol represents a dual hormonal axis that directly shapes immune balance and inflammatory responses (32). Melatonin supports anti-inflammatory cytokine production, enhances antioxidant defense mechanisms, and inhibits inflammasome activation. By contrast, cortisol in excess suppresses adaptive immune function, reduces lymphocyte proliferation, and alters cytokine profiles toward immunosuppression (33). When sleep is disrupted, melatonin levels decline while cortisol secretion often increases or becomes irregular (34). This combination produces a pathophysiological state characterized by chronic low-grade inflammation, impaired microbial regulation, and tissue breakdown (35). Such changes are particularly relevant to periodontal disease, which depends on the interaction of immune responses and microbial dysbiosis, and to xerostomia, where circadian misalignment may impair salivary gland gene expression (36).

Clinical studies reinforce this model. Sleep deprivation has been associated with elevated systemic inflammatory markers, altered salivary cytokine profiles, and increased periodontal risk (37, 38). However, most available data are cross-sectional, which limits the ability to establish causality (39). It remains unclear whether hormonal disruption initiates immune alterations or whether it simply reflects disease progression. Future research should integrate longitudinal hormone monitoring with immune phenotyping and microbial sequencing. Future studies integrating longitudinal hormone profiling with immune and microbial phenotyping will be essential to define causal hierarchies and clinically actionable signatures.

## 3 Melatonin and periodontal disease

### 3.1 Mechanistic insights

Melatonin, while best known as a circadian regulator, exerts wide-ranging biological effects that are particularly relevant to periodontal health. At the cellular level, melatonin functions as a potent antioxidant by directly scavenging free radicals and by upregulating the activity of intrinsic antioxidant enzymes, including superoxide dismutase and glutathione peroxidase (40). These actions reduce the accumulation of reactive oxygen species and protect gingival tissues from oxidative stress. In addition, melatonin suppresses key pro-inflammatory pathways. It has been shown to inhibit the activation of nuclear factor kappa B (NF-κB) and to block the assembly of the NLRP3 inflammasome, both of which are central regulators of cytokine production and inflammatory cell recruitment (41). Through these mechanisms, melatonin reduces the secretion of pro-inflammatory mediators such as interleukin-1β and tumor necrosis factor-alpha, while enhancing anti-inflammatory cytokines such as interleukin-10.

Beyond inflammation control, melatonin exerts a regulatory effect on bone metabolism. Experimental studies have demonstrated that it promotes osteoblastic differentiation and mineralization, while inhibiting osteoclastic activity and bone resorption (40). In animal models of periodontitis, local or systemic administration of melatonin attenuates alveolar bone loss and limits connective tissue destruction (42). These findings suggest that melatonin not only moderates the inflammatory cascade but also actively contributes to the preservation of alveolar bone. The convergence of antioxidant, anti-inflammatory, and bone-protective functions positions melatonin as a unique modulator of periodontal homeostasis. Despite these encouraging preclinical data, most studies remain limited to short-term or single-intervention designs, and the optimal dosage and delivery route for targeting periodontal tissues remain uncertain.

### 3.2 Experimental and clinical evidence

Accumulating evidence from experimental and translational research supports melatonin’s protective role in periodontal disease. *In vitro* studies using human gingival fibroblasts and periodontal ligament cells have shown that melatonin strengthens antioxidant defenses, decreases matrix metalloproteinase activity, and reduces inflammatory mediator release [41). These effects may limit extracellular matrix degradation and enhance the stability of periodontal connective tissues. Animal studies further demonstrate that melatonin administration reduces inflammatory cell infiltration and preserves bone height in ligature-induced periodontitis models, providing biological plausibility for clinical application (43).

Clinical investigations have extended these findings into human populations. Randomized controlled trials (RCTs) have evaluated melatonin supplementation as an adjunct to standard non-surgical periodontal therapy such as scaling and root planning (44). Several trials report significant improvements in clinical parameters, including probing depth, clinical attachment level, and gingival bleeding, particularly when follow-up is extended to three months (45). Importantly, reductions in biochemical markers of inflammation and bone turnover, such as receptor activator of NF-κB ligand and matrix metalloproteinase-8 in gingival crevicular fluid, have been observed following melatonin supplementation (42). These findings are especially noteworthy in patients with systemic comorbidities such as type 2 diabetes mellitus, who typically present with more severe inflammatory responses (46). The results suggest that melatonin may exert synergistic benefits in high-risk populations by dampening systemic oxidative stress and local periodontal inflammation. Nevertheless, variability in trial design, dosage (ranging from 3 to 10 milligrams daily), and routes of administration (oral supplementation versus local gels) complicates direct comparison across studies (43). However, current clinical studies exhibit substantial heterogeneity in both dosage and administration routes. To facilitate interpretation, existing evidence may be conceptually categorized into three patterns. First, lower systemic doses (approximately 3 mg) are generally associated with circadian regulation and systemic antioxidant effects. Second, higher systemic doses (up to 10 mg) appear to exert more pronounced anti-inflammatory and bone-protective effects, particularly in inflammatory periodontal conditions. Third, local delivery systems, such as topical gels, may provide site-specific therapeutic benefits within periodontal tissues while minimizing systemic exposure. Despite these trends, direct comparisons across studies remain limited due to variability in study design, patient populations, and outcome measures. Therefore, no universally optimal dose or administration strategy can currently be established. Future studies should aim to clarify dose-dependent mechanisms and optimize delivery approaches for clinical application.

### 3.3 Clinical potential and research gaps

Taken together, mechanistic, experimental, and clinical evidence indicates that melatonin has considerable potential as an adjunctive therapy for periodontal disease. Its combined antioxidant, anti-inflammatory, and bone-preserving effects distinguish it from conventional therapeutic agents that primarily target bacterial biofilms (47). In addition, melatonin is generally regarded as safe, with a favorable side-effect profile even at relatively high doses, which enhances its clinical feasibility (48). The potential utility of melatonin is not limited to periodontal inflammation; emerging studies suggest beneficial effects in systemic conditions such as metabolic syndrome, rheumatoid arthritis, and cancer, all of which share oxidative and inflammatory pathways with periodontitis (45). These pleiotropic benefits raise the possibility that melatonin supplementation could improve both oral and systemic health outcomes.

**Figure 2:**
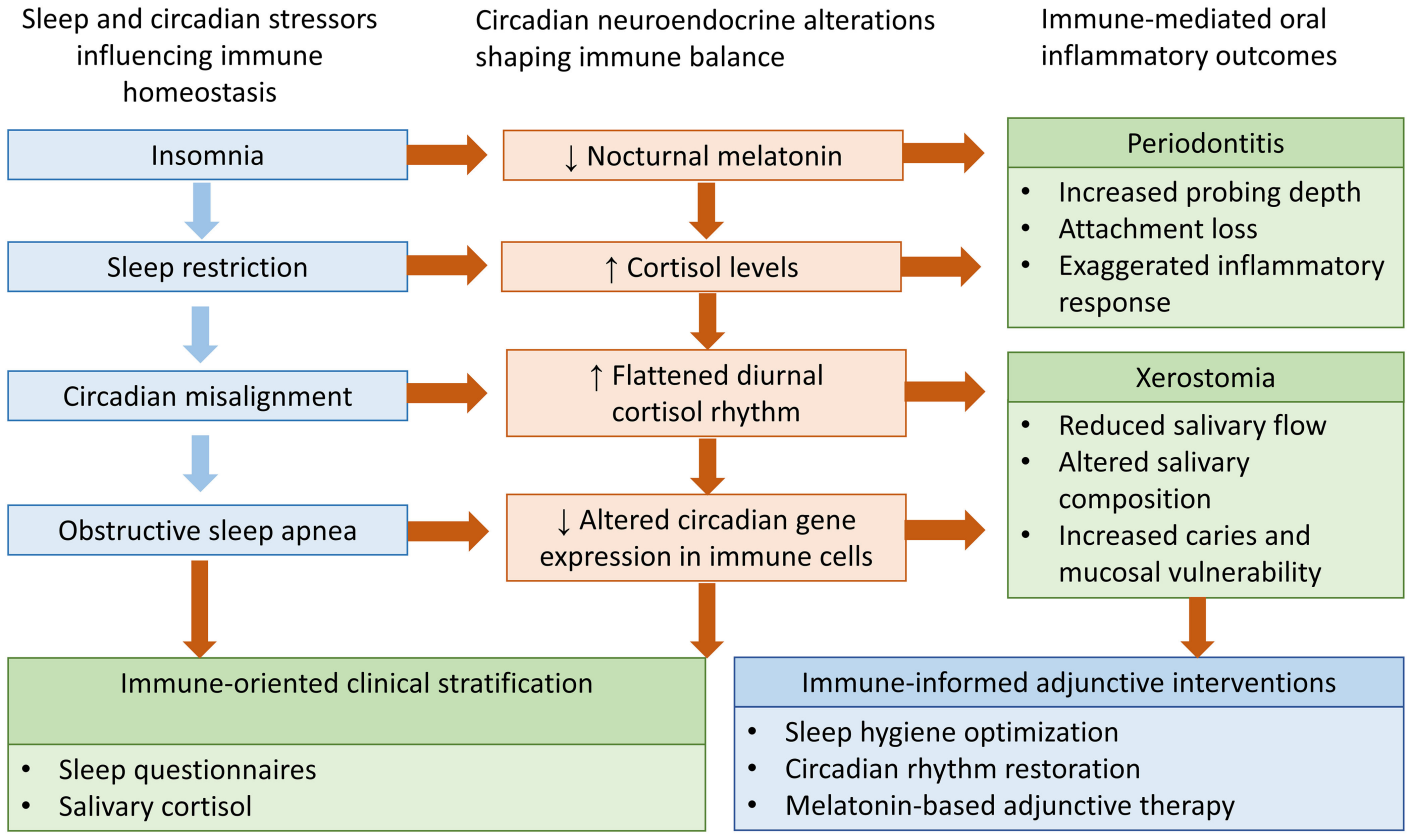
Immune-centered framework linking sleep and circadian disruption to oral inflammatory diseases. Sleep and circadian stressors disrupt neuroendocrine homeostasis, characterized by reduced nocturnal melatonin signaling, dysregulated cortisol rhythms, and altered circadian gene expression involved in immune regulation. These alterations reshape systemic and mucosal immune balance, increasing the susceptibility of periodontal tissues and salivary glands to inflammation, microbial dysbiosis, and functional impairment. The framework also highlights immune-informed clinical stratification and adjunctive interventions targeting circadian restoration.

Despite this promise, several critical gaps must be addressed before melatonin can be integrated into evidence-based periodontal practice. Current trials are relatively small and heterogeneous, limiting generalizability (46). There is no consensus on the optimal dosage, formulation, or duration of melatonin use for periodontal indications. Moreover, few studies have examined whether exogenous supplementation interferes with or complements endogenous circadian secretion patterns. Large-scale, multicenter randomized trials with standardized protocols are needed to establish efficacy and safety across diverse populations (49). Finally, mechanistic investigations into melatonin’s interaction with host microbiota and its impact on long-term periodontal stability are still lacking. Addressing these gaps will be essential to determine whether melatonin can transition from a promising adjunct to a validated therapeutic strategy in periodontal care. Collectively, these findings support an immune-centered framework in which sleep and circadian disruption drive periodontal vulnerability through coordinated neuroendocrine and immune mechanisms, as summarized in **Figure 2**.

## 4 Cortisol and periodontal disease

### 4.1 HPA axis dysregulation and mechanistic pathways

Cortisol is the principal glucocorticoid produced by the adrenal cortex under the regulation of the HPA axis. Under physiological conditions, it follows a distinct circadian pattern, with peak secretion shortly after awakening and a progressive decline throughout the day until reaching a nocturnal minimum (50). This rhythm ensures coordination of energy metabolism, stress responses, and immune surveillance (51). Sleep disruption, however, can destabilize this delicate balance. Both experimental and observational studies demonstrate that acute sleep restriction elevates evening cortisol levels and flattens diurnal rhythmicity, while chronic insomnia and shift work induce long-term HPA axis overactivation (52). These disruptions reflect maladaptive stress physiology that persists even in the absence of psychological triggers (53).

Mechanistically, elevated cortisol exerts immunosuppressive effects that compromise host defense in the oral cavity. Excess cortisol reduces neutrophil chemotaxis and phagocytic function, suppresses T-lymphocyte proliferation, and alters cytokine profiles, often diminishing protective responses while allowing pathogenic bacteria to thrive (54). Dysbiosis of the oral microbiome can therefore be promoted by cortisol-driven immune suppression, creating an environment conducive to periodontal tissue destruction (55). Furthermore, chronic cortisol excess may contribute to alveolar bone resorption by disrupting osteoblast and osteoclast dynamics (31). Collectively, these findings highlight cortisol dysregulation as a key mediator linking poor sleep with immune imbalance, microbial shifts, and periodontal breakdown (56).

### 4.2 Clinical and experimental evidence

Clinical research supports the mechanistic association between cortisol dysregulation and periodontal disease. A meta-analysis of case-control studies has shown that individuals with chronic insomnia present with moderately elevated cortisol levels compared to healthy controls, particularly when measured in serum or multiple time points throughout the day (52). Experimental sleep restriction studies confirm these findings, demonstrating a consistent rise in both adrenocorticotropic hormone and cortisol after even short periods of reduced sleep, accompanied by a blunted circadian rhythm (51). These data establish sleep loss as an independent driver of HPA axis disruption.

In periodontal populations, salivary cortisol concentrations are significantly higher in patients with periodontitis than in healthy individuals (57). Case-control studies have reported odds ratios of nearly four for elevated cortisol in periodontitis patients, with strong correlations between cortisol levels and clinical indicators such as probing depth, attachment loss, gingival index, and plaque index. Systematic reviews further support this relationship, confirming that altered salivary cortisol is consistently associated with periodontal disease severity (27). Importantly, these findings suggest that salivary cortisol could serve as a noninvasive biomarker for periodontal risk assessment. Nevertheless, methodological variability remains a major limitation. Differences in sampling protocols, circadian timing of collection, and sensitivity of assays contribute to inconsistent results across studies (58). Without standardization, the reliability of salivary cortisol as a diagnostic or prognostic marker remains uncertain (59).

### 4.3 Clinical potential and research gaps

The convergence of mechanistic and clinical evidence positions cortisol as both a mediator of periodontal tissue destruction and a potential biomarker of disease risk. Elevated cortisol not only reflects systemic stress but also appears to play an active role in immune suppression, microbial dysbiosis, and bone resorption (31). Salivary cortisol measurement is attractive because of its simplicity, noninvasive nature, and close reflection of free circulating cortisol (59). This has led to increasing interest in incorporating cortisol monitoring into periodontal risk stratification and treatment planning. However, most studies are cross-sectional, making it difficult to establish whether cortisol elevation is a causal factor, an amplifier of ongoing inflammation, or merely a byproduct of systemic disease burden (60). Future research should therefore prioritize longitudinal and interventional designs. Large cohort studies are needed to determine whether altered cortisol profiles precede the onset or progression of periodontal disease (31). Interventional trials that restore circadian rhythmicity, through behavioral sleep interventions, pharmacological chronotherapy, or stress-reduction programs, should be evaluated for their ability to normalize cortisol secretion and improve periodontal outcomes (30). Mechanistic investigations linking cortisol to host–microbiome interactions and alveolar bone metabolism are also warranted. By addressing these gaps, cortisol could move from being a correlational biomarker to a validated target for precision dentistry, bridging sleep biology with clinical periodontology (61).

## 5 Sleep and xerostomia

### 5.1 Salivary circadian regulation and mechanistic insights

Saliva production is not constant throughout the day but instead follows a circadian rhythm that is regulated by molecular clock genes expressed in major salivary glands (62). Genes such as aquaporin-5, which encodes a water channel protein essential for fluid secretion, exhibit rhythmic oscillations that govern both the quantity and the composition of saliva. Disruption of normal sleep patterns, including chronic insomnia, sleep restriction, or irregular schedules, alters the expression of these circadian regulators (63). Both human and animal studies demonstrate that circadian misalignment leads to reduced salivary flow, decreased buffering capacity, and altered electrolyte balance, including changes in calcium and magnesium ratios (64). These disruptions compromise not only lubrication but also antimicrobial defense and mineral homeostasis within the oral cavity. The biological plausibility of this mechanism highlights sleep as a systemic determinant of salivary gland function, linking disturbed circadian regulation to the onset of xerostomia (65).

### 5.2 OSA-related xerostomia and therapy-induced effects

OSA is one of the most prevalent sleep disorders associated with xerostomia. Patients frequently report morning dry mouth, which reflects both altered circadian regulation of salivary secretion and increased oral breathing during fragmented sleep (66). Cross-sectional studies show that individuals with moderate to severe OSA may present with hyposalivation, lower salivary pH, and disturbed electrolyte composition. The prevalence of xerostomia in OSA patients is consistently high, with some cohorts reporting that more than 70 percent of patients experience symptoms upon awakening. A 2021 cohort study of 30 OSA patients found that 73.3% reported xerostomia, and 20% had objectively measured hyposalivation, with a clear trend of worse symptoms in moderate to severe OSA (salivary flow: 0.28 mL/min mild vs 0.14 mL/min severe; p < 0.05) (67).

In addition to disease-related changes, treatment modalities can also influence salivary function. Continuous positive airway pressure, although effective in reducing airway obstruction, can worsen xerostomia due to continuous airflow (67, 68). Mandibular advancement devices, widely used as alternatives, may also contribute to dry mouth by altering intraoral pressure dynamics. Scoping reviews confirm that xerostomia persists in a substantial proportion of OSA patients despite therapy, although symptoms sometimes improve with long-term adherence. The dual burden of disease-related and treatment-induced xerostomia presents a clinical challenge, requiring personalized management strategies (69). **Table 1** displays biological mechanisms underlying the association between sleep disturbance and oral diseases.

**Table 1:** Mechanistic pathways linking sleep disturbance, hormonal dysregulation, and oral disease outcomes.

Sleep disturbance	Hormonal dysregulation	Key mechanism	Oral outcome	Evidence/Strength	Reference
Insomnia/sleep restriction	↑ cortisol, ↓ melatonin	HPA axis overactivation, circadian disruption, oxidative stress-driven immune dysregulation	Increased periodontal inflammation, tissue breakdown	Human studies (observational + RCTs)/Moderate-Strong	(70–73)
Shift work/circadian misalignment	Altered melatonin rhythm, dysregulated cortisol	Circadian clock disruption, immune dysregulation, circadian clock gene dysregulation	Increased inflammatory susceptibility, periodontal progression, xerostomia	Animal + human observational/Moderate	(74–76)
OSA and fragmented sleep	Intermittent cortisol surges, ↓ melatonin	Hypoxia-induced oxidative stress, HPA activation, altered salivary function	Xerostomia, hyposalivation, increased periodontal risk	Clinical (observational)/Strong	(66, 67, 69, 77, 78)
Sleep-wake disruption (salivary clocks)	Altered local melatonin signaling, cortisol exposure	Clock gene dysregulation (AQP5), impaired central-peripheral coupling	Reduced salivary flow, altered composition, xerostomia	Preclinical (animal + mechanistic)/Emerging-Moderate	(62, 79–81)
Chronic sleep disturbance (global)	Melatonin-cortisol imbalance	Sustained HPA activation, oxidative stress, microbial dysbiosis	Periodontitis progression, salivary dysfunction	Mixed evidence/Moderate	(8, 28)

Evidence strength is qualitatively categorized based on study design and consistency of findings. ↑ indicates increase; ↓ indicates decrease.

### 5.3 Mechanistic gaps and future directions in sleep-related xerostomia

Although the association between sleep disorders and xerostomia is increasingly supported by clinical and experimental evidence, major gaps remain (82). Current studies are largely cross-sectional, limiting the ability to determine causality. There is little clarity on whether xerostomia arises primarily from disrupted circadian regulation of salivary glands or secondarily from mechanical factors such as oral breathing and device-related airflow (62). In addition, the molecular pathways linking clock gene dysregulation with impaired secretion are incompletely defined. Few studies have tested whether restoring circadian alignment through behavioral or pharmacological interventions improves salivary outcomes in patients with sleep disorders (83).

Future research should therefore integrate mechanistic approaches, such as transcriptomic profiling of salivary gland clock genes, with clinical trials that evaluate sleep-centered therapies (69). Understanding the distinction between disease-related and treatment-induced xerostomia is also essential for developing targeted management strategies. Such efforts could pave the way for chronotherapy approaches in oral medicine, where timing-based interventions are used to optimize salivary gland function and alleviate xerostomia in affected populations (4).

## 6 Clinical implications and future directions

### 6.1 Clinical and translational evidence supporting sleep-immune-oral interactions

The accumulating evidence linking sleep quality, hormonal regulation, and oral health highlights sleep as a modifiable determinant in the prevention and management of periodontal disease and xerostomia (84). From a clinical standpoint, routine evaluation of sleep patterns could enrich periodontal risk assessment, particularly in patients with systemic comorbidities such as diabetes and cardiovascular disease, who already present with heightened inflammatory burden. Salivary cortisol offers promise as a practical and noninvasive biomarker of stress-related periodontal risk, correlating with disease severity and potentially guiding personalized monitoring (85). Similarly, profiling of melatonin secretion patterns may provide insight into circadian misalignment and immune imbalance in patients with unexplained periodontal inflammation or persistent xerostomia.

Adjunctive therapeutic strategies are also emerging. Melatonin supplementation, administered orally or locally, has shown clinical benefit in RCTs by improving probing depth, attachment level, and gingival bleeding, with pronounced effects in high-risk groups (41). Interventions aimed at restoring healthy cortisol rhythms, such as behavioral sleep modification or chronotherapy, may mitigate cortisol-driven tissue destruction, although these remain largely experimental. Together, these findings suggest that integrating sleep assessment and hormonal biomarker monitoring into dental practice could transform periodontal care from a reactive model to a preventive, precision-oriented approach (44, 86). While mechanistic insights derived from experimental models strongly implicate circadian hormonal dysregulation in immune-mediated oral pathology, translational validation in human populations is critical. Accumulating evidence from observational, case-control, cross-sectional, and interventional studies has begun to establish consistent associations between sleep disturbances, neuroendocrine alterations, and clinically relevant oral inflammatory outcomes. Representative studies across different methodological designs are summarized in **Table 2**.

**Table 2:** Summary of clinical and experimental studies linking sleep-related circadian stressors, neuroendocrine alterations, and immune-mediated oral inflammatory outcomes.

Study design	Sleep stressor	Neuroendocrine change	Oral outcome	Key interpretation
Observational (human)	Insomnia	↓Nocturnal melatonin	Periodontitis	suggests that impaired circadian regulation may contribute to periodontal inflammation
Case-control	OSA	↑Salivary cortisol	Periodontitis	supports the role of cortisol as a stress-related biomarker linked to periodontal severity
Cross-sectional	OSA	Altered circadian rhythm	Xerostomia	indicates that circadian disruption may impair salivary gland function and mucosal immunity
RCTs	Sleep restriction	↑Evening cortisol	Inflammatory response	provides evidence supporting a causal link between sleep loss and systemic immune dysregulation

Collectively, these studies reveal convergent patterns linking sleep disruption to suppressed nocturnal melatonin signaling, dysregulated cortisol rhythms, and increased susceptibility to periodontal inflammation and salivary dysfunction, while also highlighting heterogeneity in study design, biomarker assessment, and clinical outcomes.

The table highlights representative study designs and provides interpretative summaries to facilitate comparison across evidence types. ↑ indicates increase; ↓ indicates decrease.

### 6.2 Research gaps and future priorities

Despite emerging clinical and translational evidence, several critical knowledge gaps remain (4). Most studies examining the relationship between sleep disturbance, hormonal dysregulation, and oral disease are heterogeneous and predominantly cross-sectional, with inconsistent biomarker sampling, variable sleep phenotyping, and limited immune profiling. Such designs constrain causal inference and hinder determination of whether circadian hormonal imbalance initiates oral pathology or merely reflects disease progression (87). Longitudinal cohort studies are therefore required to clarify temporal relationships between sleep disruption, melatonin and cortisol dysregulation, and periodontal or salivary outcomes (31, 84).

Beyond observational research, interventional trials that directly test whether improving sleep quality or restoring circadian alignment attenuates periodontal inflammation and xerostomia are essential to validate causal mechanisms (84, 88). In parallel, mechanistic studies should further elucidate how circadian clock gene dysregulation within periodontal and salivary tissues contributes to immune imbalance and disease susceptibility. The integration of multi-omic approaches, including transcriptomic and microbiome profiling, may uncover hormonal–immune signatures that enhance risk stratification and refine predictive models (62).

From a clinical perspective, sleep quality represents a clinically relevant yet underrecognized determinant of oral health (89). Sleep disturbances such as insomnia, circadian misalignment, and OSA are highly prevalent and frequently coexist with chronic inflammatory conditions (90). Given their bidirectional interactions with endocrine and immune regulation, these sleep disorders should be considered systemic modifiers of periodontal disease and salivary gland dysfunction rather than isolated lifestyle factors. Incorporating basic sleep screening into dental practice may therefore offer substantial benefit. Simple assessments of sleep duration, sleep quality, and symptoms of sleep-disordered breathing could aid in identifying patients at elevated risk for inflammation-driven periodontal breakdown or persistent xerostomia (91). Such an approach aligns with the broader shift toward comprehensive patient profiling in oral medicine, particularly for individuals who exhibit disproportionate periodontal destruction or refractory xerostomia despite adequate local therapy (92). Hormonal biomarkers associated with sleep regulation represent promising tools for advancing precision oral healthcare. Salivary cortisol, owing to its noninvasive collection and established association with stress and circadian rhythm disruption, has emerged as a candidate marker for stress-related periodontal disease susceptibility (93). Sustained elevation or flattening of diurnal cortisol rhythms may reflect maladaptive HPA axis activity, impair host immune defense, and promote microbial dysbiosis. Altered melatonin secretion patterns may similarly signal compromised antioxidant and immunomodulatory capacity, predisposing periodontal and salivary tissues to inflammatory injury (94). Although hormonal testing is not yet routine in dental settings, refinement of sampling protocols and reference ranges may facilitate future clinical translation.

Therapeutically, sleep-centered interventions represent an underexplored adjunctive strategy for oral disease management. Behavioral approaches aimed at improving sleep duration, regularity, and circadian alignment may indirectly reduce systemic inflammation and enhance immune resilience. Pharmacological interventions, such as melatonin supplementation, have demonstrated anti-inflammatory and antioxidant effects in experimental and clinical contexts (95), although substantial heterogeneity remains regarding dosing, timing, and routes of administration (96). Whether exogenous melatonin can replicate the temporal and tissue-specific actions of endogenous secretion within periodontal and salivary tissues remains unresolved. Interventions targeting cortisol dysregulation are even less developed; strategies that restore circadian rhythmicity, including sleep hygiene optimization, light exposure modulation, and stress management, may mitigate the detrimental effects of sustained cortisol elevation on periodontal tissues (97). Chronotherapeutic approaches, which align interventions with biological rhythms, represent a promising but largely unexplored direction in oral healthcare (98).

At a broader conceptual level, recognition of sleep as a modifiable systemic factor in oral disease pathogenesis expands the framework of periodontology and oral medicine. Integrating sleep assessment, hormonal biomarkers, and circadian biology into research and clinical practice may facilitate individualized, immune-informed prevention and treatment strategies. Addressing sleep disturbances has the potential not only to improve oral health outcomes but also to position dentistry within a systems-based model of precision medicine (83, 99, 100).

## 7 Discussion

Sleep and circadian regulation have emerged as central modulators of immune homeostasis across multiple organ systems, yet their relevance to oral inflammatory diseases has only recently gained systematic attention. In this review, we integrate clinical, translational, and mechanistic evidence to propose that disrupted sleep reshapes oral immune equilibrium through coordinated neuroendocrine dysregulation, primarily involving the melatonin and cortisol axes. This immune-centered perspective provides a unifying framework linking sleep disturbance to periodontal disease and xerostomia, while highlighting broader implications for oral medicine and systemic inflammatory health.

### 7.1 Clinical applications and translational significance

From a translational standpoint, recognizing sleep disturbance as a biologically meaningful modifier of oral inflammation expands current paradigms in periodontal and salivary disease management. Accumulating evidence, as outlined in earlier sections, indicates that sleep-related hormonal alterations influence immune surveillance, oxidative stress responses, and host–microbial interactions within periodontal tissues and salivary glands. These effects suggest that sleep quality and circadian integrity should be considered alongside traditional local risk factors when assessing susceptibility to inflammation-driven oral disease. Incorporating sleep assessment into routine dental practice may offer tangible clinical benefit. Simple screening tools evaluating sleep duration, sleep quality, and symptoms of sleep-disordered breathing could assist in identifying individuals at heightened risk for disproportionate periodontal breakdown or persistent xerostomia. Such an approach aligns with emerging trends in oral medicine that emphasize comprehensive patient profiling and recognition of systemic modifiers underlying localized disease manifestations.

Building upon these observations, hormonal biomarkers associated with sleep regulation represent promising tools for advancing precision oral healthcare. Salivary cortisol, owing to its noninvasive collection and established links to stress and circadian disruption, has been proposed as a candidate marker for identifying patients susceptible to stress-related periodontal disease progression. Sustained elevation or flattening of diurnal cortisol rhythms may reflect maladaptive HPA axis activity, with downstream consequences for immune regulation and microbial balance in the oral cavity. Altered melatonin secretion patterns may similarly signal compromised antioxidant and immunomodulatory capacity, potentially predisposing periodontal and salivary tissues to inflammatory injury. Although hormonal testing is not yet routine in dental settings, continued refinement of sampling protocols and interpretation frameworks may facilitate future clinical translation.

### 7.2 Research gaps and future priorities

Despite growing evidence, several critical knowledge gaps remain in understanding the relationship between sleep disturbance, hormonal dysregulation, and oral disease progression. Most available studies are heterogeneous and predominantly cross-sectional, limiting causal inference regarding the temporal relationship between sleep disturbance, hormonal dysregulation, and oral disease progression. As a result, it remains unclear whether circadian hormonal imbalance initiates periodontal and salivary pathology or primarily reflects downstream consequences of established inflammation. Longitudinal cohort studies are therefore essential to clarify temporal dynamics linking sleep disruption, melatonin and cortisol dysregulation, and immune-mediated oral outcomes. Such studies should incorporate standardized sleep phenotyping, time-resolved biomarker sampling, and longitudinal immune profiling to disentangle cause-effect relationships. Beyond observational research, interventional trials that directly manipulate sleep quality or circadian alignment, rather than focusing solely on pharmacological modulation, are needed to validate causal mechanisms and therapeutic potential.

At the mechanistic level, further investigation is required to elucidate how circadian clock gene disruption within periodontal tissues and salivary glands alters immune responsiveness and tissue resilience. Advances in molecular chronobiology, combined with multi-omic approaches such as transcriptomics and microbiome profiling, may enable identification of tissue-specific rhythmic signatures predictive of disease susceptibility or therapeutic response. Integrating these molecular insights with clinical biomarkers could support development of immune-informed risk stratification strategies and personalized intervention frameworks.

### 7.3 Broader immunological implications and integrative perspective

Beyond their relevance to oral health, the concepts discussed in this review underscore the broader immunological significance of sleep and circadian regulation in mucosal immunity. Oral tissues represent a unique interface between systemic immune regulation and continuous microbial exposure, rendering them particularly sensitive to neuroendocrine perturbations. Disruption of circadian hormonal signaling may therefore amplify inflammatory vulnerability not only locally but also across interconnected mucosal and systemic immune networks.

Recognizing sleep as a modifiable systemic factor in oral disease pathogenesis expands the conceptual scope of periodontology and oral medicine. Integrating sleep biology, hormonal biomarkers, and immune regulation into both research and clinical practice may facilitate a transition toward more holistic, systems-based models of patient care. Addressing sleep disturbance has the potential not only to improve periodontal and salivary outcomes but also to position oral healthcare professionals as contributors to broader precision medicine initiatives that bridge immune, endocrine, and behavioral health domains.

Building upon these perspectives, the findings synthesized in this review further support a multi-level framework in which sleep and circadian disruption influence oral health through coordinated neuroendocrine–immune mechanisms. As summarized in **Table 1**, disturbed sleep induces a shift toward a cortisol-dominant, pro-inflammatory state accompanied by suppression of melatonin-mediated protective pathways, resulting in oxidative stress, immune dysregulation, and increased tissue vulnerability. Complementing this mechanistic model, the evidence summarized in **Table 2** demonstrates converging patterns across observational, clinical, and experimental studies, linking sleep-related hormonal alterations to clinically relevant oral outcomes, including periodontitis and xerostomia.

Together, these findings suggest that sleep disturbance should not be viewed merely as a behavioral or lifestyle factor, but rather as a biologically active systemic modifier of oral inflammatory disease. The integration of mechanistic insight with cross-level evidence highlights a consistent axis of neuroendocrine imbalance that bridges circadian disruption with immune-mediated oral pathology. This conceptual framework provides a foundation for future research aimed at identifying causal pathways, refining biomarker-based risk stratification, and developing circadian-informed therapeutic strategies. Ultimately, incorporating sleep and circadian biology into oral medicine may facilitate a transition toward more precise, system-oriented approaches in the prevention and management of inflammatory oral diseases.
